# Strength criterion of rock mass considering the damage and effect of joint dip angle

**DOI:** 10.1038/s41598-022-06317-1

**Published:** 2022-02-16

**Authors:** Yugen Li, Huimei Zhang, Min Chen, Xiangzhen Meng, Yanjun Shen, Hui Liu, Yani Lu

**Affiliations:** 1 School of Architecture Engineering, Yulin University, Yulin, 719000 Shaanxi China; 2 School of Science, Xi’an University of Science and Technology, Xi’an, 710054 Shaanxi China; 3 College of Architectural and Civil Engineering, Xi’an University of Science and Technology, Xi’an, 710054 Shaanxi China; 4 College of Geology and Environment, Xi’an University of Science and Technology, Xi’an, 710054 Shaanxi China; 5 School of Civil Engineering, Hubei Engineering University, Xiaogan, 432000 Hubei China

**Keywords:** Geology, Petrology, Solid Earth sciences

## Abstract

It is highly significant to theoretically assess the effect, under load, of initial stress and structure on the mass damage of rock mass. In this reported study, first a multi-factor coupling damage constitutive model under the action of joint-load was established by fully considering the non-uniformity, anisotropy and initial structure of a rock mass based on the Weibull distribution and D-P criterion. The relationship between the damage evolution and joint angle in the rock mass was elaborated. Then, a jointed rock mass strength criterion was built in line with the D–P criterion and the limit state of rock mass failure by the method of multivariate function total differential as based on the constitutive model. The results showed that the established constitutive model was in good agreement with the test results, which accurately reflected the damage characteristics of jointed rock mass during the entire loading process. The initial damage value of the rock mass increased with increasing joint dip angles, and the damage evolution of the jointed rock mass could be divided into the initial, stable, accelerated and failure damage stages. Comparing the results of this approach with other methods it was found that the strength criterion better reflected the effects of minimum principal stress *σ*_3_, volume stress *σ*_*m*_, shear stress *J*_2_^1/2^ and joint dip angle *β* on rock mass strength than other existing strength criteria, which showed that the proposed method offered important guiding principles for the engineering practice.

## Introduction

The rock mass is a geologic body composed of rock blocks and other weak structural planes such as joints, bedding, as well as faults in practical engineering, which will have an important effect on its macro-mechanical properties and the stability of surrounding rock, and sometimes even need to be reinforced by rockbolt or other technology to improve its safety in practice^1,2^. The jointed rock mass, which is cut by a large number of relatively small structural planes and has more complex internal structure. Generally, these joints are macroscopic defects distributed in different directions of the rock mass and affect its cracking mechanism^3,4^.

The intact rock is usually used to make a persistent joint with initial shearing damage to simulate the failure process jointed rock mass under load. In this way, the rock mass will be affected by the initial joint damage and load damage two damage factors, so that the material cohesion is progressive. That is to say, the deformation and failure of jointed rock mass is caused by the coupling and accumulation of these two damage effects. Thence, it is necessary to effectively describe the failure process of rock mass according to the constitutive model and obtain its ultimate failure state beginning with the essential characteristics of damage. Moreover, establishing a strength criterion that considers the damage characteristics and joint inclination effects is of great significance.

Currently, a large number of domestic and foreign researchers have analyzed the mechanical properties of jointed rock mass using both theoretical and experimental methods. Yang et al.^5^ analyzed the internal relationships among the joint dip angle, rock strength characteristics and failure modes of rock mass based on the laboratory mechanics tests. Liu et al.^6^ and Yang et al.^7^ studied the influence of joint conditions on the deformation and failure of rock mass specimens. Their conclusions are important to guide for engineering practice.

Constructing the constitutive models of jointed rock mass has always been a hot issue in engineering and academic community. Kyoya et al.^8^ introduced the damage mechanics theory into the study of jointed rock mass for the first time, and established its constitutive model by using the effective stress method, which providing a new idea for the study of jointed rock mass. Chen et al.^9^ defined the damage variables by joint frequency and established the micro-planar constitutive model of jointed rock mass based on the method of combining parallel and equal-spaced joints with rock elastic matrix. Yuan et al.^10,11^, Wang et al.^12^ and Liu et al.^13^ have also done a lot of work on this focus, but the results have some limitations.

Numerical simulation is also an effective method to study the load damage of jointed rock mass. Bahaaddini et al.^14^ and Meng et al.^15^ reproduced the failure process of rock mass with different joint dip angles using different numerical calculation methods, and analyzed the impact of dip angles on the macro-mechanical properties of rock mass. Cao et al.^16^ discussed the mechanical properties of jointed rock mass under unidirectional stress from the macro scale using the software PFC2D. Ma et al.^17^ studied the influence of confining pressure on representative elementary volume size of jointed rock mass by the method of three-dimensional discrete element numerical, and the conclusion is more consistent with the actual project.

Strength criterion is the key to the design and nonlinear numerical calculation in geotechnical engineering, which has greatly promoted the development of rock mechanics. Domestic and foreign researchers have established many valuable strength criteria based on different theories and methods. AL-Ajmi et al.^18^ established Mogi–Coulomb criterion by analyzing the variation relationship between fitting parameters and shear strength parameters on the basis of linear Mogi criterion; Hoek–Brown^19^ proposed the failure criteria for the jointed rock mass, Ramamurthy et al.^20^ obtained the nonlinear strength criterion of jointed rock mass based on the analysis of triaxial compression test results. Pietruszczak et al.^21^ established a strength criterion of rock mass that considers both the fabric tensor and stress tensor by introducing the fabric tensor. Zhang et al.^22^, Li et al.^23^ modified Hoek–Brown criterion and verified it through a large number of test data. Xie et al.^24^ analyzed the rock failure mechanism according to the rock failure energy principle and established the rock energy failure strength criterion. Zhou et al.^25^ established a unified energy yield criterion by analyzing the relationship between the two energies during rock yield from the perspective of energy. However, some of these results are obtained based on the isotropic strength theory and static load, which can only describe the failure conditions that the stress or strain should meet, but fail to reveal the deformation and failure process of the rock mass and do not agree well with engineering practice.

In conclusion, the existing damage constitutive model and strength criteria are of great impotence to engineering practice, but they have their shortcomings. For example, first, the mechanical properties of jointed rock mass were mainly obtained by the experimental methods, but few studies have theoretically described the deformation process of rock under load, or they cannot effectively characterize mechanical behavior and reflect the randomness of rock micro-element damage during rock mass failure. Second, most of the strength criteria have been established based on the shear strength theory or isotropic strength theory, which cannot accurately reflect the influence of minimum principal stress *σ*_3_ and volume stress *σ*_*m*_ on the strength. Moreover, these approaches fail to build the strength criterion based on the nonlinear response of the coupling effects of joint and load damage to reveal the macroscopic deformation and failure characteristics of rock mass, that is, it cannot reflect the essential characteristics of damage mechanics.

In this reported study, a joint-load multi-factor coupling damage constitutive model under the three-dimensional stress state was established based on the continuous distribution function of Weibull distribution and D–P criterion. These were combined with the geometric conditions at the peak point of the stress–strain curve to describe the random damage process of rock mass under load by fully considering the influence of inhomogeneity, anisotropy and initial structure damage (joint) on the strength of rock mass. The selected model parameters can better reflect the changing of ductility, brittleness and strength of the rock mass, which has a more clear physical meaning than other parameters included in existing model determined by the fitting method. Then, the effect of initial structural (joint) on the model parameters, total damage and strength of rock was analyzed according to the test results. Finally, the jointed rock mass strength criterion was established in line with D–P criterion, the limit state of rock mass failure and the constitutive model by the method of multivariate function total differential, and its rationality was verified by the test results.

## Jointed rock mass damage constitutive model

### Damage evolution equation

**Figure 1:**
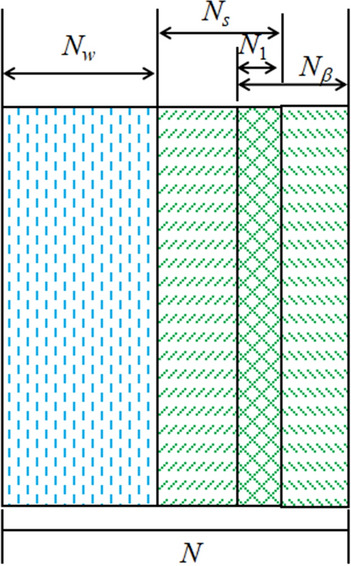
Axial elements diagram of rock.

There is local initial damage in the rock mass due to the presence of a joint surface. During the loading process, the joint and load will gradually weaken the cohesive force of the material through different mechanical mechanisms, and this will induce the mutual influence and mutual coupling of the two kinds of damage. In the process of deformation and failure of jointed rock under load, the rock elements can be divided into undamaged elements *N*_*w*_, joint initial damaged elements *N*_*β*_, load damaged elements *N*_*s*_ and coupling damaged elements *N*_1_, as shown in Fig. 1.

The establishment of the damage evolution equation begins with the definition of the joint initial damage. Assuming that the total number of micro-elements in the rock mass is *N*, the number of damaged ones formed by the prefabricated joints is *N*_β_, and the initial damaged variable of the joints *D*_*β*_ can be defined as:

1 $$ D_{\beta } = \frac{{N_{\beta } }}{N} $$

Meanwhile, the rock mass is further damaged by the action of loading. Assuming that the number of micro-elements damaged by loading is *N*_*s*_*,* the number of micro-elements damaged by the coupling effect of joint and loading is *N*_1_, and the load damage variable of jointed rock mass can be defined as:

2 $$ D_{s} = \frac{{N_{s} - N_{1} }}{{N - N_{\beta } }} $$

Finally, the total damage variable of jointed rock mass under loading can be defined as follow based on the final damage degree.

3 $$ D_{t} = \frac{{N_{\beta } + N_{s} - N_{1} }}{N} $$

Substituting Eqs. (1) and (2) into Eq. (3), the total damage variable can be expressed by the initial joint damage variable *D*_*β*_ and load damage variable *D*_*s*_ as:

4 $$ D_{t} = D_{s} + D_{\beta } - D_{s} D_{\beta } $$

In Eq. (4), *D*_*β*_ reflects the influence degree of macroscopic joints on the damage of rock mass, and *D*_*s*_ reflects the trend of continuous damage deterioration when rock mass is subjected to load. This equation also shows that there is a nonlinear relation between the total damage variable *D*_*t*_, the initial joint damage variable *D*_*β*_ and the load damage variable *D*_*s*_. In general, the two types of damage action aggravate the total damage to the rock when they work together, but its coupling effect weakens the total damage (shown as − *D*_*s*_*D*_*β*_).

**Figure 2:**
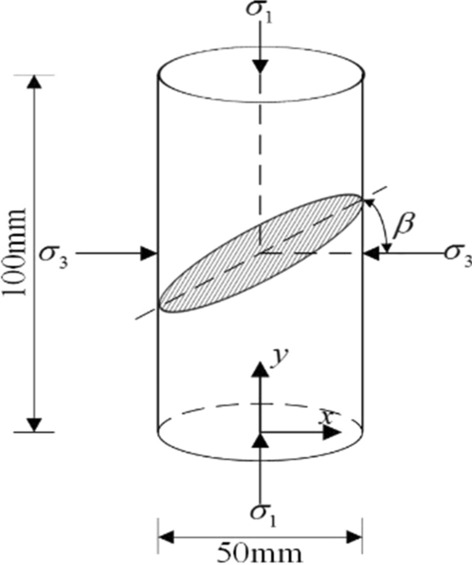
Definition of joint dip angle *β.*

For the jointed rock mass, a macroscopic defect is present that is cut by different dip angle joint surface, which causes deterioration of the mechanical properties and results in a strong response to the deformation and strength characteristics of the rock. It is well known that this degree of deterioration of the material can be characterized by the macro-mechanical property response of rock according to the theory of macroscopic phenomenological damage mechanics. Therefore, the elastic modulus is used to measure the initial damage of jointed rock mass, and the initial damage variable *D*_*β*_ can be defined as follow:

5 $$ D_{\beta } = {1} - \frac{{E_{\beta } }}{{E_{{0}} }} $$

where *β* is the joint dip angle (the angle between the minimum principal stress σ_3_ and the joint plane as shown in Fig. 2), *E*_*β*_ is elastic modulus of the rock with different joint inclinations, and *E*_0_ is elastic modulus of the intact rock.

Meanwhile, the jointed rock mass exhibits non-uniformity in micro-structure and anisotropy in macro-depression under the influence of joints, which allows for a probabilistic distribution of micro-elements strength inside the material. When loaded, more micro-defects are generated, split and expanded into new macro-defects, and the failure of micro-element exhibits randomness. Therefore, we often use a statistical law to express the nonlinear macroscopic properties of materials caused by the accumulation of random failure of micro-elements.

As a continuous distribution function, Weibull distribution accurately reflects the random damage process of rock-elements under load and was used to build the damage constitutive model recently^26–28^. Assuming that the strength of rock micro-elements obeys the Weibull distribution, the load damage variable can be expressed as:

6 $$ D_{s} = \int_{{0}}^{F} {P(F)dF = } 1 - e^{{ - \left( {\frac{{F^{*} }}{{F_{{0}} }}} \right)^{m} }} $$

where $F^{*}$ is the distribution variable used to characterize the intensity of micro-elements, *m* and *F*_0_ are Weibull statistical distribution parameters related to the mechanical properties of materials.

Substituting Eqs. (5) and (6) into Eq. (4), the total damage variable *D*_*t*_ can be expressed as:

7 $$ D_{t} = {1} - \frac{{E_{\beta } }}{{E_{{0}} }}e^{{ - \left( {\frac{{F^{*} }}{{F_{{0}} }}} \right)^{m} }} $$

### Establishment of damage constitutive model

According to the equivalent strain principle proposed by Lemaitre^29^, the damage constitutive model of jointed rock mass can be written as:

8 $$ \left[ \sigma \right]{ = }\left[ {\sigma^{*} } \right]\left( {{1} - D} \right) = \left[ E \right]\left[ \varepsilon \right]\left( {{1} - D} \right) $$

where [*σ*] is the nominal stress matrix, [σ*] is the effective stress matrix, *D* is the damage variable, [*E*] is the elastic matrix, and [*ε*] is the strain matrix.

Combining Eqs. (7) and (8), we can obtain the damage constitutive equation of jointed rock mass under triaxial conditions as shown:

9 $$ \sigma_{{1}} { = }E_{\beta } \varepsilon_{{1}} e^{{ - \left( {\frac{{F^{*} }}{{F_{0} }}} \right)^{m} }} { + 2}\mu_{\beta } \sigma_{{3}} $$

10 $$ \left( {{1} - \mu_{\beta } } \right)\sigma_{{3}} = E_{\beta } \varepsilon_{{3}} e^{{ - \left( {\frac{{F^{*} }}{{F_{0} }}} \right)^{m} }} { + }\mu_{\beta } \sigma_{{1}} $$

where *σ*_1_, *σ*_3_ are the nominal stresses in the direction of axial and lateral, respectively. *μ*_*β*_ is the Poisson's ratio of rock mass with different joint inclinations.

According to the Drucker–Prager (D–P) criterion^30^, F^*^ can be expressed as:

11 $$ F^{*} = \alpha I^{*} + J_{2}^{*1/2} $$

where $I_{{1}}^{ * }$ and $J_{{2}}^{ * }$ are the first invariant of effective stress tensor and second invariant of effective stress lateral, which can be written as Eqs. (12) and (13), respectively. *α* is the constant related to the internal friction angle *φ* of rock mass calculated by the circumscribed circle of the inner corner of Mohr–Coulomb^31^. It can be expressed as Eq. (14).

12 $$ I^{*} = \sigma_{{1}}^{*} + \sigma_{{2}}^{*} + \sigma_{{3}}^{*} $$

13 $$ J_{2}^{*} = \frac{1}{6}\left[ {\left( {\sigma_{{1}}^{*} - \sigma_{{2}}^{*} } \right)^{2} + \left( {\sigma_{{2}}^{*} - \sigma_{{3}}^{*} } \right)^{2} + \left( {\sigma_{{3}}^{*} - \sigma_{{1}}^{*} } \right)^{2} } \right] $$

14 $$ \alpha { = }\frac{{{\text{2sin}}\varphi }}{{\sqrt 3 \left( {3 + {\text{sin}}\varphi } \right)}} $$

where *σ*_1_^*^ is the effective stresses in axial direction, *σ*_2_^*^and *σ*_3_^*^ are the effective stresses in lateral direction.

Combining Eqs. (11), (12) and (14), Eqs. (9) can be further expressed as:

15 $$ F^{*} = \alpha I_{1}^{{{*} }} + J_{2}^{{{*1.2} }} = \left( {\alpha I_{1} + J_{2}^{1/2} } \right)\frac{{E_{0} \varepsilon_{1} }}{{\sigma_{1} - 2\mu_{\beta } \sigma_{3} }} $$

where $I_{{1}}$ and $J_{{2}}$ are the first invariant of nominal stress tensor and second invariant of nominal stress lateral, which can be written as:

16 $$ I = \sigma_{{1}} + \sigma_{{2}} + \sigma_{{3}} $$

17 $$ J_{{2}} = \frac{1}{6}\left[ {\left( {\sigma_{{1}} - \sigma_{{2}} } \right)^{2} + \left( {\sigma_{{2}} - \sigma_{{3}} } \right)^{2} + \left( {\sigma_{{3}} - \sigma_{{1}} } \right)^{2} } \right] $$

The theoretical values of parameters *m* and *F*_0_ used in jointed rock mass constitutive model can be obtained by the stress–strain curve at the peak point with the following extreme conditions:

① $$\varepsilon_{{1}} { = }\varepsilon_{{\text{c}}}$$, $$\sigma_{{1}} { = }\sigma_{{\text{c}}}$$
② $$\varepsilon_{{1}} { = }\varepsilon_{{\text{c}}}$$, $$\frac{{\partial \sigma_{{1}} }}{{\partial \varepsilon_{{1}} }}{ = 0}$$

Combining Eqs. (9), (10), (15) and the two geometric extreme conditions ① and ②, the model parameters *m* and *F*_0_ can be expressed as Eqs. (18) and (19), finally:

18 $$ m = \frac{{1}}{{\ln \frac{{E_{\beta } \varepsilon_{{\text{c}}} }}{{\sigma_{{\text{c}}} - 2\mu_{\beta } \sigma_{3} }}}} $$

19 $$ F_{0} = \left( {\alpha \frac{{\sigma_{{\text{c}}} { + }2\sigma_{3} }}{{\sigma_{{\text{c}}} - 2\mu_{\beta } \sigma_{3} }}{ + }\frac{{\sigma_{{\text{c}}} { - }\sigma_{3} }}{{\sqrt {3} \left( {\sigma_{{\text{c}}} - 2\mu_{\beta } \sigma_{3} } \right)}}} \right)E_{{0}} \varepsilon_{{\text{c}}} \left( m \right)^{1/m} $$

As can be seen in Eqs. (18) and (19), the model parameters are in line with the elasticity modulus, Poisson's ratio, confining pressure, peak strength, peak strain and other mechanical characteristic parameters of the jointed rock mass. Therefore, they reflect the internal damage mechanism and describe the mechanical behavior of rock mass.

Substituting Eqs. (18) and (19) into Eqs. (7) and (9), we can finally obtain the damage evolution equation and constitutive model of jointed rock mass.

### Discussion of model parameters

#### Physical meaning of model parameters

**Figure 3:**
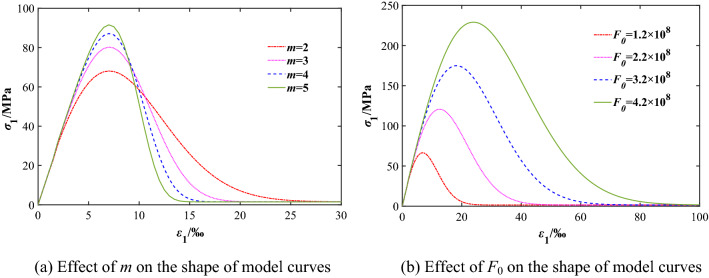
Effect of model parameters on the shape of model curves.

The discussion starts by clarifying the physical meaning of model parameters *m* and *F*_0_. Previous research results^32,33^ were used to analyze the influence of model parameters on the mechanical properties of rock mass with the joint angle of 15° and confining pressure of 3 MPa. Figure 3 shows the influence of *m* and *F*_0_ on the *σ*_1_–*ε*_1_ curve of rock mass according to Eqs. (16) and (17).

As can be seen in Fig. 3a, the curve changes little in the linear elastic stage before the peak point, but the slope of plastic yield section and post-peak softening section becomes steeper, and the rate of descent of the curve after the peak point increases with the increase of model parameter *m*, which means that there is an increasing of the brittleness and a weakening of ductility of jointed rock mass. That is to say, the parameter *m* reflects the ductility and brittleness characteristics of the jointed rock and describes the intensity distribution concentration of micro-element in rock mass (the homogeneity of mechanical properties).

As can be seen in Fig. 3b that the intensity and strain of the curve at peak point increases significantly with little change of the curve shape before the peak point. However, the slope of the curve is basically fixed with the increase of model parameter *F*_0_. After the peak point, the curve moves approximately to the right. It means that *F*_0_ is the average value of micro-elements strength in macro statistics, which reflects the strength of the jointed rock mass.

#### Influential effect analysis of model parameters

**Figure 4:**
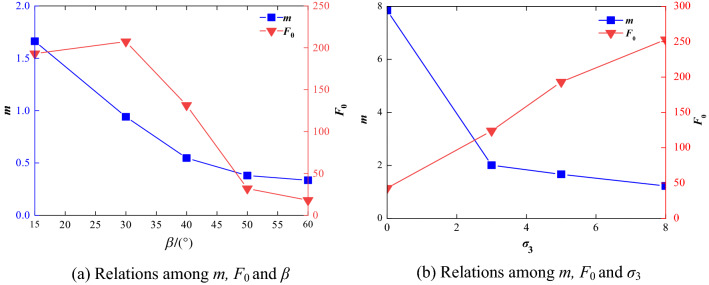
Effect of confining pressure and joint dip angle on model parameters.

The deformation and strength properties of rock mass is mainly determined by its mineral composition, structural surface characteristics and confining pressure. The model parameters, can characterize their effects indirectly. Analyzing the variation of model parameters *m* and *F*_0_ with joint inclination angle *β* and confining pressure *σ*_3_ is very important for revealing the strength and failure characteristics of a rock mass. The relation between *β* and *m*, *F*_0_, as well as the relation between *σ*_3_ and *m, F*_0_ is shown in Fig. 4 according to the previous research results^32,33^ with *σ*_3_ = 5 MPa and *β* = 15°.

As can be seen in Fig. 4a, both *m* and *F*_0_ decrease with the increase of joint dip angle when the confining pressure is fixed (σ_3_ = 5 MPa), which indicates that the bearing capacity of the rock is reduced and its ductility continuously increases. In general, the maximum values of *m* and *F*_0_ appear at the angle of 15° and 30°, respectively, but the minimum values all appears at 60°, which indicates a significant degree of ductility and the lowest peak strength of the rock mass.

Figure 4b shows that as the confining pressure changes from 0 to 3 MPa, the value of *m* first decreases rapidly and continuously as the confining pressure increases, and then it gradually decreases. This indicates that the brittleness of the jointed rock mass decreases with the increase of confining pressure. When the confining pressure reached 5 MPa, the rock mass exhibited obvious ductility characteristics, which can be clearly seen from the variation of parameter *m* (the curve is almost horizontal)*.* In the case of parameter *F*_0_, its value increases with increasing confining pressure, and the change in the amplitude of the curve gradually decreases, which indicates that both the ability to resist deformation and the strength of jointed rock mass is gradually enhanced by the increase in the confining pressure, and the enhancement in strength is more significant.

According to the above discussion, the ductility and brittleness changes and the strength characteristics of rock mass revealed by the variation of parameters in the initial stage. The values *m* and *F*_0_ are consistent with the experimental results reported in the literature^32,33^, which proves that the model parameters established in this paper is correct.

## Damage characteristic analysis of jointed rock mass

### Law of damage evolution

The deformation process of a rock mass is nonlinear because of the development and evolution of macroscopic joints and micro-defects under load. Hence, it is necessary to explore the structural damage and the evolutionary path of macro mechanical properties induced by damage to reveal the essential characteristics of rock mass damage, and reflect the nonlinear progressive failure behavior of materials.

**Figure 5:**
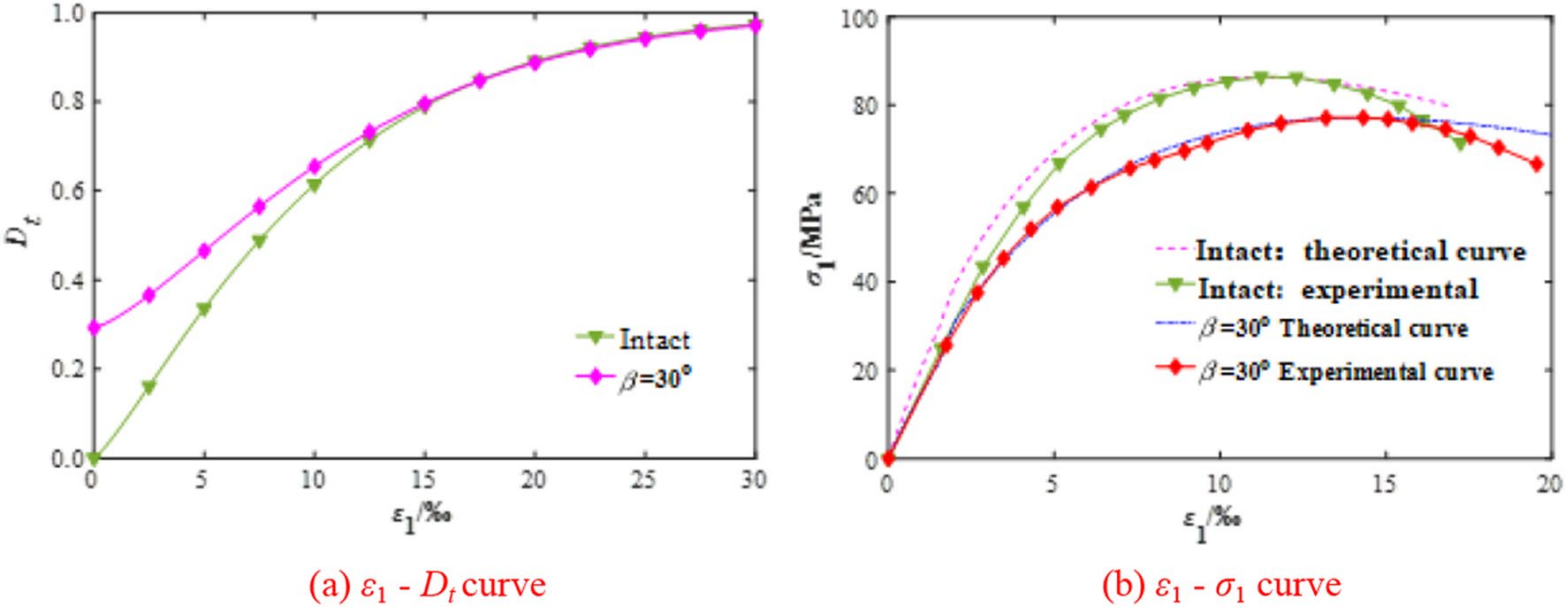
Correspondence of the damage evolution curve and stress–strain curve.

Previous test results of the intact and rock mass reported in the literature^32,33^ were selected to verify the accuracy of the established model according to Eq. (7), and the results are shown in Fig. 5. As shown in Fig. 5a, the damage evolution section of the intact rock was smaller than that of jointed rock mass during the deformation stage. The damage evolution curves of the two types of rock samples both show an “S” shape with the increase of strain, which reflects the various stages of deformation and failure of the rock mass when loaded. The jointed rock mass, however, was more easily deformed because of the existence of a joint surface. Specifically, for the intact rock, the damage evolution curve starts from the point (0, 0), which means that there is no initial damage.

In the case of the jointed rock, its damage evolution curve does not pass through point (0, 0) due to the initial damage of joint. In general, both damage evolution curves of the two type rock samples exhibit a concave shape at the initial damage stage. The reason for this is that the deformation is mainly caused by the compression density and closure of the original defect, and the friction generated by the closed surface limits its sliding and the initiation and propagation of the new micro-crack, so the value of total damage variable *D*_*t*_ is small. In other words, the rock mass is in the stage of compaction and linear elastic deformation at this stage, so the *ε*_1_*–σ*_1_ curve shows linear relation.

Figure 5b shows that the presence of a joint that weakens the strength of rock mass compared with intact rock. For the intact rock, the peak strength is 94.3 MPa and the peak strain is 0.0112, while for the jointed rock mass, the peak strength is reduced to 85.2 MPa and the peak strain is increased to 0.0142, which indicates that the strength of rock mass is weakened and the ductility is enhanced when joints are present. Figure 5 also shows that the mechanical behavior of rock deformation reflected by the damage evolution curve corresponds to both the test and theoretical results of *ε*_1_*–σ*_1_, which indicates the rationality of the damage model established in this paper.

**Figure 6:**
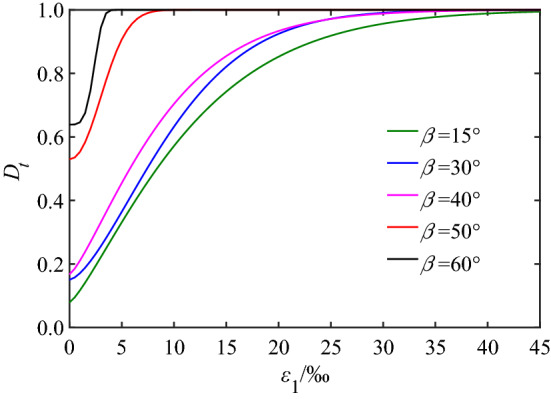
Damage evolution of rock mass under different joint dip angle.

Moreover, the relationship between the damage evolution and joint dip angle was analyzed taking the joint dip angles of 15°, 30°, 40°, 50^o^ and 60°, confining pressure of 5 MPa as an example based on the existing reports^32,33^, and the result is shown in Fig. 6. These data appear to show that the damage value corresponding to *ε*_1_ = 0 is the initial damage of the rock mass caused by the joint. It can be seen that the initial damage value of rock mass increased with increasing joint dip angles. The maximum value appeared at the angle of 60°, and its damage value rapidly approached 1 when the strain value increased by less than 5‰. The main reason for this result was that the rock mass in this condition was close to failure due to structural damage.

Meanwhile, the change of the damage evolution curve of jointed rock masses has obvious similarity with the continuous increase of strain, and the process can be divided into four typical stages: the initial, stable, accelerated and damage failure stages.

In the initial damage stage, the macro-joints and micro-cracks in the rock mass tend to be the same under the load, the closed surfaces do not extend or expand, the damage evolution curve is approximately horizontal, and the rock mass is in the compaction and elastic deformation stage.

In the stable damage stage, initiated by the continuous action of loading, the micro-fracture develops, the rock particles and joint surface slip and dislocate resulting in unrecoverable deformation and nonlinear growth of damage, so the rock mass enters the plastic yield stage. The nonlinear law of the *ε*_1_–*σ*_1_ curve shown in Fig. 5 illustrated this result very well.

In the accelerated damage stage, there is a continuous expansion of cracks in rock mass caused by the stress concentration at the end of joint. The primary defects develop rapidly, the interaction between cracks intensifies, the large-scale cracks connect, which produces the forming of new cracks and fracture surfaces, so the damage accelerates, and the bearing capacity of rock mass decreases.

After entering the damage failure stage, the damage evolution curve gradually flattens and the value of total damage variable *D*_t_ gradually approaches 1. At this point, the stress is released instantaneously, resulting in the failure of rock mass.

### Structural effects of damage

There are many key feature points in the deformation process of jointed rock mass under load, which can characterize the mechanical response law of rock mass. Therefore, the macro-mechanical response of jointed rock mass can be predicted by analyzing the damage of the key feature points in the failure process of jointed rock mass. The damage value at the peak point can reflect the damage value corresponding to the maximum bearing capacity in the process of material deformation and failure. So, the variation rule of rock damage at the peak point with joint dip angle is discussed afterward.

**Figure 7:**
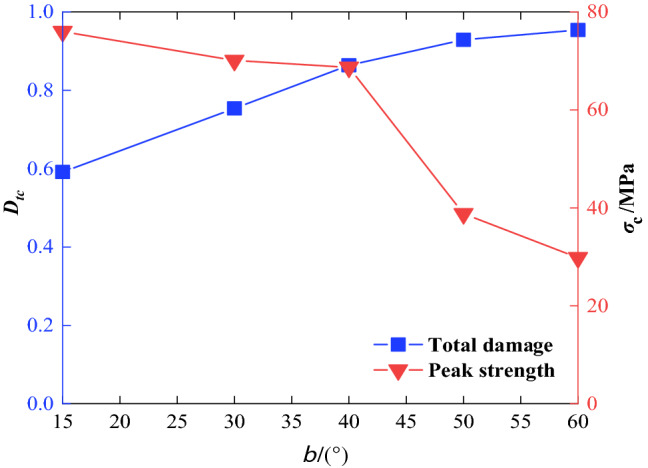
Relations among the peak strength, total damage value and joint dip angle.

Based on Eqs. (7) and (9), the experimental data contained in the literature^32,33^ were again used to analyze the relationship among the total damage at the peak point of rock mass, the joint dip angle and the confining pressure. Using a confining pressures of 5 MPa as examples, Fig. 7 shows the variation law of total damage and strength at the peak point along with the joint dip angle.

As can be seen in Fig. 7, there are significant differences in the degree of damage and peak strength of rock mass with different joint conditions. With the increase of joint dip angle (15° ≤ *β* ≤ 60°), the total damage value generally increases, while the peak strength decreases nonlinearly. Meanwhile, the variation of total damage and strength is consistent. When the dip angles are 15° and 30°, the total damage value is relatively small, and the corresponding peak strength of rock mass is relatively high. At this time, the joint dip is smaller than the friction angle in rock body, and the strength of rock mass depends on the strength of its matrix. When the dip angle of 60°, the total damage reaches the maximum value and the corresponding rock mass has the lowest strength. At this point, the stress on the joint surface is basically in a state of limited equilibrium, and the shear slip failure occurs along the joint surface of rock mass, which results in an inability to resist the failure and damage. This is basically consistent with rock mass failure angle (*β* = *π/4* + *Φw/*2) calculated by the single structural plane theory^34^, that is, the strength of rock mass is mainly controlled by the strength of joint plane.

Using confining pressure *σ*_3_ = 5 MPa as an example for quantitative analysis, when the joint dip angle is 15°, the total damage value is 0.5915, and it increases to 0.7538, 0.8641, 0.9291 when the angle increasing to 30°, 40°, 50° with the growth rate of 27.44%, 46.09% and 57.08%, respectively. When the joint dip angle increases to 60°, the total damage value is 0.9538, and the damage variable is close to 1. At this point, the damage caused by the structure plane in the rock mass is close to failure, and its resistance to failure is extremely poor.

The above discussion shows that the variation rule of damage calculated according to our model corresponds to the change of the peak strength of jointed rock mass, which is in agreement with the experimental results described in the literature^32,33^. That is to say, it is reasonable to describe the damage evolution behavior of a jointed rock mass by using the model proposed in this paper.

### Verification of the constitutive model

**Figure 8:**
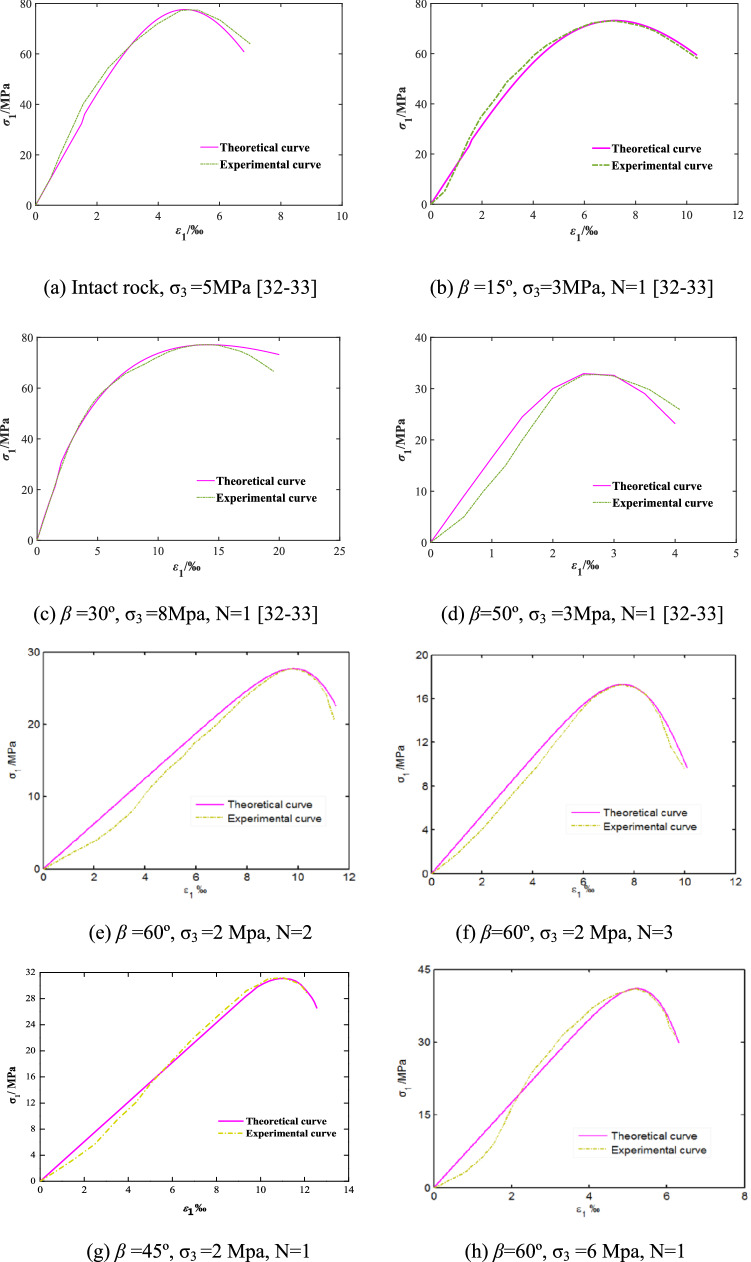
Comparison of test and theoretical constitutive curves of rock mass.

Four groups of rock-like materials reported in literature^32,33^, four groups of red sandstone from our tests results, total eight groups of representative rock mass with size of Ф 50 mm × 100 mm, different joint dip angles and confining pressures were taken as examples to verify the correctness of the model. For the rock-like materials, the intact sample and jointed sample with one persistent joint dip angles of 15°, 30^o^ and 50^o^ were compressed by the triaxial testing machine with the confining pressure of 5 MPa, 3 MPa, 8 MPa and 3 MPa, respectively. During loading, the force control method was used at the beginning, and when the load reached 1 kN, it was changed to displacement control with the speed of 3 mm/min, and the results are shown in Fig. 8a–d.

While for the red sandstone, samples with one, two and three persistent joints in length of 10 mm, dip angles of 45° and 60° were compressed by the triaxial testing machine with the confining pressure of 2 MPa and 6 MPa, respectively. During loading, the confining pressure was applied at a rate of 0.05 MPa/s, followed by the axial loading with the displacement rate of 0.05 mm/s, and the results are shown in Fig. 8e–i.

As can be seen in Fig. 8, the curves obtained by the constitutive model of jointed rock mass are in good agreement with both the reported data in references^32,33^ and our experimental results, which closely describes the mechanical behavior of deformation and failure of the rock mass with different joint conditions and confining pressures. Moreover, it obeys the law of rock mass strength affected by stress state and structural effect.

## Damage strength criterion of jointed rock mass

### Establishment of strength criteria

In summary, a constitutive model of jointed rock mass under complex stress states has been constructed using a unified functional form, which can describe the internal mechanism and structural characteristics of jointed rock mass deformation and failure, and has laid a solid foundation for the establishment of strength criteria. According to the deformation and failure curve (σ_1_–ε_1_ curve) of jointed rock mass, the extreme point of the maximum principal stress is found, and the relation between the stress components in jointed rock mass material under the limit state can be obtained. Based on the total differential law of multi-function, *σ*_1_ and *σ*_3_ are regarded as the functions of *ε*_1_, *ε*_3_ and joint angle *β* with the forms as shown in Eqs. (20) and (21) according to Eqs. (9) and (10):

20 $$ d\sigma_{{1}} = \frac{{\partial \sigma_{{1}} }}{{\partial \varepsilon_{{1}} }}d\varepsilon_{{1}} + \frac{{\partial \sigma_{{1}} }}{{\partial \varepsilon_{{3}} }}d\varepsilon_{{3}} + \frac{{\partial \sigma_{{1}} }}{\partial \beta }d\beta $$

21 $$ d\sigma_{{3}} = \frac{{\partial \sigma_{{3}} }}{{\partial \varepsilon_{{1}} }}d\varepsilon_{{1}} + \frac{{\partial \sigma_{{3}} }}{{\partial \varepsilon_{{3}} }}d\varepsilon_{{3}} + \frac{{\partial \sigma_{{3}} }}{\partial \beta }d\beta $$

In Eq. (15), the rock micro-element strength of F* is expressed by *ε*_1_. In fact, it can also be expressed by *ε*_3_ as follows:

22 $$ F^{*} = F_{1}^{*} = \alpha I_{1}^{{{*} }} + J_{2}^{{{*1/2} }} = \left( {\alpha I_{1} + J_{2}^{1/2} } \right)\frac{{E_{0} \varepsilon_{1} }}{{\sigma_{1} - 2\mu_{\beta } \sigma_{3} }} $$

23 $$ F^{*} = F_{3}^{*} = \alpha I_{1}^{{{*} }} + J_{2}^{{{*1/2} }} = \left( {\alpha I_{1} + J_{2}^{1/2} } \right)\frac{{E_{0} \varepsilon_{3} }}{{\left( {1 - \mu_{\beta } } \right)\sigma_{3} - \mu_{\beta } \sigma_{1} }} $$

Taking the total differential on both sides of Eqs. (9) and (10), we can get:

24 $$ d\sigma_{{1}} = \frac{{\partial \sigma_{{1}} }}{{\partial \varepsilon_{{1}} }}d\varepsilon_{{1}} + \frac{{\partial \sigma_{{1}} }}{{\partial F_{{1}}^{*} }}dF_{{1}}^{*} + \frac{{\partial \sigma_{{1}} }}{{\partial F_{{0}} }}dF_{{0}} + \frac{{\partial \sigma_{{1}} }}{\partial m}dm + \frac{{\partial \sigma_{{1}} }}{\partial \beta }d\beta + 2\mu_{\beta } d\sigma_{{3}} $$

25 $$ \left( {{1} - \mu_{\beta } } \right)d\sigma_{{3}} = \frac{{\partial \sigma_{{3}} }}{{\partial \varepsilon_{{3}} }}d\varepsilon_{{3}} + \frac{{\partial \sigma_{{3}} }}{{\partial F_{{3}}^{*} }}dF_{{3}}^{*} + \frac{{\partial \sigma_{{3}} }}{{\partial F_{{0}} }}dF_{{0}} + \frac{{\partial \sigma_{{3}} }}{\partial m}dm + \frac{{\partial \sigma_{{3}} }}{\partial \beta }d\beta + \mu_{\beta } d\sigma_{{1}} $$

According to Eqs. (22) and (23), dF* included in Eqs. (24) and (25) can be further fully differentiated with a form containing only *dσ*_1_, *dσ*_3_, *dε*_1_, *dε*_3_ and *dβ*:

26 $$ dF_{{1}}^{*} = \frac{{\partial F_{{1}}^{*} }}{{\partial \varepsilon_{{1}} }}d\varepsilon_{{1}} + \frac{{\partial F_{{1}}^{*} }}{{\partial \sigma_{{1}} }}d\sigma_{{1}} + \frac{{\partial F_{{1}}^{*} }}{{\partial \sigma_{{3}} }}d\sigma_{{3}} + \frac{{\partial F_{{1}}^{*} }}{\partial \beta }d\beta $$

27 $$ dF_{{3}}^{*} = \frac{{\partial F_{{3}}^{*} }}{{\partial \varepsilon_{{3}} }}d\varepsilon_{{3}} + \frac{{\partial F_{{3}}^{*} }}{{\partial \sigma_{{1}} }}d\sigma_{{1}} + \frac{{\partial F_{{3}}^{*} }}{{\partial \sigma_{{3}} }}d\sigma_{{3}} + \frac{{\partial F_{{3}}^{*} }}{\partial \beta }d\beta $$

Assuming that *F*_0_ and *m* are the functions of *σ*_3_ and the joint inclination *β*, respectively, then:

28 $$ dF_{{0}} = \frac{{\partial F_{{0}} }}{{\partial \sigma_{{3}} }}d\sigma_{{3}} + \frac{{\partial F_{{0}} }}{\partial \beta }d\beta $$

29 $$ dm = \frac{\partial m}{{\partial \sigma_{3} }}d\sigma_{3} + \frac{\partial m}{{\partial \beta }}d\beta $$

Substituting Eqs. (26), (27), (28) and (29) into Eqs. (24) and (25), we can get:

30 $$ {\text{C}}_{{1}} d\sigma_{{1}} + {\text{C}}_{{2}} d\varepsilon_{{1}} + {\text{C}}_{{3}} d\sigma_{{3}} + {\text{C}}_{{4}} d\beta = 0 $$

31 $$ {\text{D}}_{1} d\sigma_{{1}} + {\text{D}}_{2} d\varepsilon_{3} + {\text{D}}_{3} d\sigma_{3} + {\text{D}}_{4} d\beta = 0 $$

where $${\text{C}}_{{1}} = \frac{{\partial \sigma_{{1}} }}{{\partial F_{{1}}^{*} }}\frac{{\partial F_{{1}}^{*} }}{{\partial \sigma_{{1}} }} - 1$$, $${\text{C}}_{{2}} = \frac{{\partial \sigma_{{1}} }}{{\partial \varepsilon_{{1}} }} + \frac{{\partial \sigma_{{1}} }}{{\partial F_{{1}}^{*} }}\frac{{\partial F_{{1}}^{*} }}{{\partial \varepsilon_{{1}} }}$$, $${\text{C}}_{{3}} = \frac{{\partial \sigma_{{1}} }}{{\partial F_{{1}}^{*} }}\frac{{\partial F_{{1}}^{*} }}{{\partial \sigma_{{3}} }}{ + }\frac{{\partial \sigma_{{1}} }}{{\partial F_{{0}} }}\frac{{\partial F_{{0}} }}{{\partial \sigma_{{3}} }}{ + }\frac{{\partial \sigma_{{1}} }}{\partial m}\frac{\partial m}{{\partial \sigma_{{3}} }} + 2\mu_{\beta }$$,

$${\text{C}}_{{4}} = \frac{{\partial \sigma_{{1}} }}{{\partial F_{{1}}^{*} }}\frac{{\partial F_{{1}}^{*} }}{\partial \beta }{ + }\frac{{\partial \sigma_{{1}} }}{{\partial F_{{0}} }}\frac{{\partial F_{{0}} }}{\partial \beta }{ + }\frac{{\partial \sigma_{{1}} }}{\partial m}\frac{\partial m}{{\partial \beta }} + \frac{{\partial \sigma_{{1}} }}{\partial \beta }$$, $${\text{D}}_{{1}} { = }\frac{{\partial \sigma_{{3}} }}{{\partial F_{{3}}^{*} }}\frac{{\partial F_{{3}}^{*} }}{{\partial \sigma_{{1}} }} + \mu_{\beta }$$, $${\text{D}}_{{2}} { = }\frac{{\partial \sigma_{{3}} }}{{\partial \varepsilon_{{3}} }} + \frac{{\partial \sigma_{{3}} }}{{\partial F_{{3}}^{*} }}\frac{{\partial F_{{3}}^{*} }}{{\partial \varepsilon_{{3}} }}$$,

$${\text{D}}_{{3}} { = }\frac{{\partial \sigma_{3} }}{{\partial F_{{3}}^{*} }}\frac{{\partial F_{{3}}^{*} }}{{\partial \sigma_{{3}} }} + \frac{{\partial \sigma_{{3}} }}{{\partial F_{{0}} }}\frac{{\partial F_{{0}} }}{{\partial \sigma_{{3}} }} + \frac{{\partial \sigma_{{3}} }}{\partial m}\frac{\partial m}{{\partial \sigma_{{3}} }}{ + }\mu_{\beta } - 1$$, $${\text{D}}_{{4}} { = }\frac{{\partial \sigma_{{3}} }}{{\partial F_{{3}}^{*} }}\frac{{\partial F_{{3}}^{*} }}{\partial \beta } + \frac{{\partial \sigma_{{3}} }}{{\partial F_{{0}} }}\frac{{\partial F_{0} }}{\partial \beta } + \frac{{\partial \sigma_{{3}} }}{\partial m}\frac{\partial m}{{\partial \beta }}{ + }\frac{{\partial \sigma_{{3}} }}{\partial \beta }$$.

Combining Eqs. (20), (30) and (31), we can get Eq. (32) according to the extreme value condition ②, and which can be simplified as shown in Eq. (33).

32 $$ \frac{{\partial \sigma_{{1}} }}{{\partial \varepsilon_{{1}} }} + \frac{{\partial \sigma_{{1}} }}{{\partial F_{{1}}^{*} }}\frac{{\partial F_{{1}}^{*} }}{{\partial \varepsilon_{{1}} }}{ = 0} $$

33 $$ F^{*} { = }F_{0} \left( m \right)^{ - 1/m} $$

Combining Eqs. (22) and (33), it can be obtained as:

34 $$ \alpha I_{1} + J_{2}^{1/2} = \frac{{\sigma_{1} - 2\mu_{\beta } \sigma_{3} }}{{E_{0} \varepsilon_{1} }}F_{0} \left[ \frac{1}{m} \right]^{1/m} $$

Substituting Eqs. (33) and (34) into Eq. (9), we finally obtain the relation between different partial quantities of jointed rock mass under the limit state:

35 $$ \alpha I_{{1}} + J_{{2}}^{{1/2}} = \frac{{E_{\beta } }}{{E_{0} }}F_{{0}} m^{{\left( { - 1/m} \right)}} {\text{e}}^{{\left( { - \frac{1}{m}} \right)}} $$

Equation (35) is the strength criterion of rock mass we established considering the effect of joint dip angle based on D–P criterion and constitutive model combining with the extreme value condition at the peak point of the stress–strain curve, which can reflect the relation between the internal stress parameters under the ultimate state of rock mass with different joint dip angles.

### Verification of strength criterion

**Figure 9:**
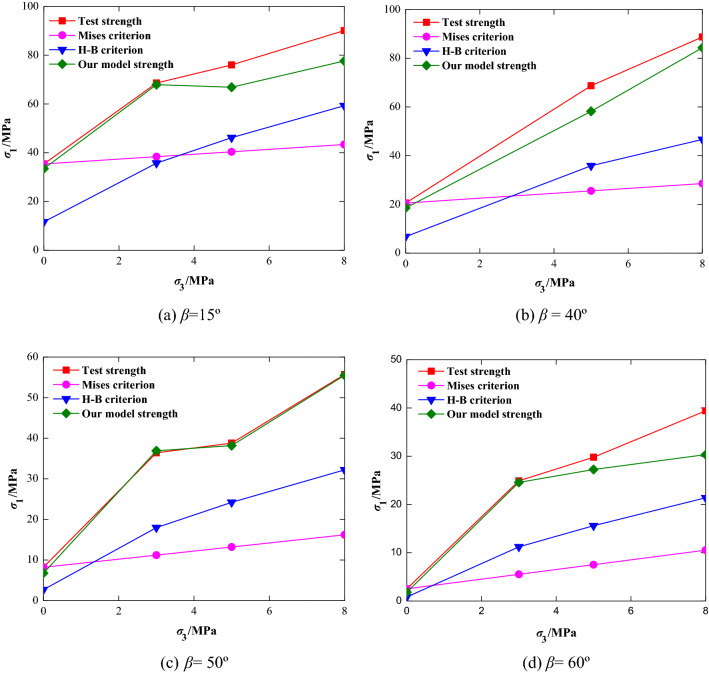
Comparison of strength curves of rock mass with different joint dip angle.

In order to verify the validity and rationality of the strength criteria of the jointed rock mass as constructed in this paper, experimental data from the literature^32,33^ were employed in calculations using Eq. (35), and the theoretical curves of four representative joint dip angles were drawn and compared with test results reported in the literature^32,33^, the Hoek–Brown criterion and Misses criterion, as shown in Fig. 9.

As can be seen in Fig. 9, the test strength curve of the rock was higher than that determined by Hoek–Brown's, and the deviation was more significant as the confining pressure increased. The possible reasons for this result can be attributed to two aspects. On the one hand, the Hoek–Brown strength criterion is mainly applicable to the broken rock mass or fractured rock mass with a wide range of discontinuities. Therefore, the estimated strength of jointed rock mass in literature^32,33^ is low. On the other hand, Hoek–Brown criterion requires determination of geological strength index (GSI) based on the quantitative analysis of surface roughness of rock mass, distribution rate of discontinuities, degree of weathering and other factors, which is greatly affected by the observer’s subjectivity^35^. Since the mechanical parameter indices are relatively consistent with sandstone, and the rock samples contain only a single joint plane, so the Hoek–Brown criterion parameters GSI = 80, α = 0.5 and *m*_*i*_ = 18 are selected in our study according to literature^36,37^.

The Mises criterion curve, however, is significantly lower than that of the test strength curve, and the difference increases with the increase in confining pressure, which is mainly caused by the physical meaning of the Mises criterion. According to the Mises criterion, under certain deformation conditions, when the elastic deformation energy of the change in shape per unit volume reaches a certain limit, the yield failure of the material begins. However, the maximum stress is not necessarily reached when yielding, and the yield point of most rock is not obvious and difficult to determine during the test, which results in the great difference between theoretical and experimental results.

Compared with Hoek–Brown and Mises criterion, the theoretical curve of the jointed rock mass strength criterion established in this study is in good agreement with the experimental curve, only with the largest error at the dip angle of 60°. Meanwhile, as the confining pressure is increased, the ultimate strength of a jointed rock mass gradually increases, and the growth rate is generally slower, which reflects the curvilinearity of the strength envelope of the jointed rock mass. This indicates that the strength criterion curve of a jointed rock mass as established in this paper accurately reflects the relationship between the stress components in the ultimate stress state of rock mass under various jointed conditions.

## Conclusions

In this reported study, a constitutive model considering the effect of joint dip angle was established based on the Weibull distribution and D–P criterion, this represents the first attempt to model the effect of joints on rock mass damage under load. Next, the strength criterion was built using the multivariate function total differential method and compared with other models to verify its veracity. The main conclusions of this study are as follows:

1. The constitutive model established based on the Weibull distribution and D–P criterion agrees well with the test results, which can better describe the damage process of jointed rock mass under load. In addition, the model results encompass the effect of initial structural defects (joints) on the strength of the rock mass.
2. The initial damage value of rock mass increases with increasing joint dip angles, and the maximum value appears at the joint dip angle of 60°, the reason is that the joints weaken the structural of rock mass. The damage evolution of jointed rock mass can be divided into the initial, stable, accelerated and failure damage of four typical stages, which is in good corresponding relation with its macro-mechanical and deformation response during the whole loading process.
3. The strength criterion established based on the multivariate function total differential method can well reflect the influences of stress state *σ*_3_, volume stress *σ*_*m*_ (*σ*_*m*_ = *I*_1_/3), shear stress *J*_2_^1/2^ and joint dip angle *β* on rock mass strength, which is more in line with the engineering practice than other existing strength criteria.

## Nomenclature

*m*: Statistical distribution parameter of Weibull
*F*_0_: Statistical distribution parameter of Weibull
*N*_*w*_: Number of undamaged elements
*N*_*β*_: Number of joint initial damaged elements
*N*_*s*_: Number of load damaged elements
*N*_1_: Number of coupling damaged elements
*N*: Number of total micro-elements
*D*_*β*_: Joints damaged variable
*D*_*s*_: Load damage variable
*D*_*t*_: Total damage variable
*I*_1_^*^: First invariant of effective stress tensor
*J*_2_^***^: Second invariant of effective stress deviation
*I*_1_: First invariant of nominal stress tensor
*a*: D–P criterion parameter
*ε*_c_: Peak strain
*F*_1_^⁎^: Axial distribution variable
*σ*_1_: Nominal axial stress
*ε*_1_: Nominal axial strain
*σ*_1__*_: Effective stress
*σ*_2__*_: Effective stress
*σ*_3__*_: Effective stress
*β*: Joint dip angle
*φ*: Internal friction angle
[*σ*]: Nominal stress matrix
[*σ**]: Effective stress matrix
[*E*]: Elastic matrix
[*ε*]: Strain matrix
*μ*_*β*_: Poisson's ratio
*J*_2_: Second invariant of nominal stress bias
*σ*_c_: Peak stress
*F*^⁎^: Distribution variable
*F*_3_^⁎^: Lateral distribution variable
